# The SAKK cancer-specific geriatric assessment (C-SGA): a pilot study of a brief tool for clinical decision-making in older cancer patients

**DOI:** 10.1186/1472-6947-13-93

**Published:** 2013-08-23

**Authors:** Kerri M Clough-Gorr, Lea Noti, Peter Brauchli, Richard Cathomas, Marius R Fried, Gillian Roberts, Andreas E Stuck, Felicitas Hitz, Ulrich Mey

**Affiliations:** 1 Institute of Social and Preventive Medicine (ISPM), University of Bern, Finkenhubelweg 11, Bern CH-3012, Switzerland; 2 Section of Geriatrics, Boston University School of Medicine, Boston Medical Center, Boston, MA, USA; 3 Division of Geriatrics, Department of General Internal Medicine, Inselspital University Hospital and University of Bern, Bern, Switzerland; 4 Swiss Group for Clinical Cancer Research, SAKK Coordinating Centre, Bern, Switzerland; 5 Kantonsspital Graubünden, Chur, Switzerland; 6 Oncology-Haematology Department, Kantonsspital St. Gallen, St. Gallen, Switzerland; 7 Department of Internal Medicine, Hematology, Medical Oncology, and Pneumology, University Medical Center of the Johannes Gutenberg University, Mainz, Germany

**Keywords:** Assessment, Cancer-specific geriatric assessment, Decision-making, Geriatric assessment, Older cancer patients, Older adults

## Abstract

**Background:**

Recommendations from international task forces on geriatric assessment emphasize the need for research including validation of cancer-specific geriatric assessment (C-SGA) tools in oncological settings. This study was to evaluate the feasibility of the SAKK Cancer-Specific Geriatric Assessment (C-SGA) in clinical practice.

**Methods:**

A cross sectional study of cancer patients **≥**65 years old (N = 51) with pathologically confirmed cancer presenting for initiation of chemotherapy treatment (07/01/2009-03/31/2011) at two oncology departments in Swiss canton hospitals: Kantonsspital Graubünden (KSGR N = 25), Kantonsspital St. Gallen (KSSG N = 26). Data was collected using three instruments, the SAKK C-SGA plus physician and patient evaluation forms. The SAKK C-SGA includes six measures covering five geriatric assessment domains (comorbidity, function, psychosocial, nutrition, cognition) using a mix of medical record abstraction (MRA) and patient interview. Five individual domains and one overall SAKK C-SGA score were calculated and dichotomized as below/above literature-based cut-offs. The SAKK C-SGA was evaluated by: patient and physician estimated time to complete, ease of completing, and difficult or unanswered questions.

**Results:**

Time to complete the patient questionnaire was considered acceptable by almost all (≥96%) patients and physicians. Patients reported slightly shorter times to complete the questionnaire than physicians (17.33 ± 7.34 *vs.* 20.59 ± 6.53 minutes, p = 0.02). Both groups rated the patient questionnaire as easy/fairly easy to complete (91% *vs.* 84% respectively, p = 0.14) with few difficult or unanswered questions. The MRA took on average 8.32 ± 4.72 minutes to complete. Physicians (100%) considered time to complete MRA acceptable, 96% rated it as easy/fairly easy to complete. Individual study site populations differed on health-related characteristics (excellent/good physician-rated general health KSGR 71% *vs.* KSSG 32%, p = 0.007). The overall mean C-SGA score was 2.4 ± 1.12. Patients at KSGR had lower C-SGA scores (2.00 ± 1.19 *vs.* 2.81 ± 0.90, p = 0.009) and a smaller proportion (28% *vs.*65%, p = 0.008) was above the C-SGA cut-off score compared to KSSG.

**Conclusions:**

These results suggest the SAKK C-SGA is a feasible practical tool for use in clinical practice. It demonstrated discriminative ability based on objective geriatric assessment measures, but additional investigations on use for clinical decision-making are warranted. The SAKK C-SGA also provides important usable domain information for intervention to optimize outcomes in older cancer patients.

## Background

Cancer is considered an age-related disease of increasing public health concern due to worldwide aging populations. The World Health Organization (WHO) reports a projected continued rise in cancer mortality with an estimated 13.1 million cancer deaths worldwide in 2030 [1]. The majority of cancer patients presenting for cancer treatment are older (>65 years old). Older cancer patients are often diagnosed at later stages, undertreated, and rarely included in clinical trials [2,3]. In fact, evidence on cancer treatment is mainly generated in younger cancer patients. Because aging is an individualized process older cancer patients represent a heterogeneous group requiring specific management–an oncological and research challenge [2,4].

Comprehensive multidimensional geriatric assessment has been shown in general population studies to be a promising tool of assessment domains that capture a range of patient factors resulting in an individualized intervention-plan for optimizing clinical management and health outcomes [5-7]. Similarly, cancer-specific geriatric assessment (C-SGA) with multiple assessment domains can aid in identifying, managing and potentially correcting (through intervention) problems that might specifically interfere with cancer treatment [8,9]. For over a decade C-SGA has been a growing field of cancer-related research. Much of the earlier literature only allowed for analysis of indirect evidence and clinical opinion supporting the use of C-SGA [10]. More recent publications include a range of study designs as well as reviews that have expanded C-SGA specific knowledge. (for example [11-15]) Moreover, recommendations from the International Society of Geriatric Oncology (SIOG) task force on geriatric assessment and the European Organization for Research and Treatment of Cancer (EORTC) Elderly Task Force have underscored the need for additional research including validation of C-SGA tools in oncological settings. [16,17] Despite growing evidence additional studies to determine C-SGA’s ability to predict relevant outcomes such as choice of treatment, treatment tolerance, treatment completion, survival, quality of life, and comparative effectiveness to physician judgment are still needed [13,16,18-24]. Incorporating C-SGA into clinical trials of older cancer patients could provide such evidence, establish an objective measure for inclusion into clinical trials, and further advance the knowledgebase accelerating translation of use into evidence-based practice. Evidence from use of C-SGA in clinical trial settings has been increasing but does not directly inform on feasibility of implementation in daily oncological practice [24-26]. In clinical practice the ease and time to perform a C-SGA is a vital to whether or not it will be adopted in regular practice and requires specific investigation.

The Swiss Group for Clinical Cancer Research (Schweizerische Arbeitsgemeinschaft für Klinische Krebsforschung [SAKK]) developed the SAKK C-SGA specifically for use in clinical trials including older cancer patients as well as daily oncological practice. The SAKK C-SGA includes six standard geriatric assessment measures covering five geriatric assessment domains (comorbidity, function, psychosocial, nutrition, and cognition) using a mix of medical record abstraction (MRA) and patient interview (described in Table 1). The individual measures are brief, reliable, valid, and predictive of morbidity and mortality in geriatric patients [27-33]. The measures have been well studied in the geriatric-oncology and geriatric assessment literature [34]. The SAKK C-SGA was developed based on an extensive literature search and expert clinical advice to be brief and easy to implement in busy clinical settings (i.e. low physician and patient burden appropriate for clinical practice as well as clinical trials). The patient questionnaire alone was previously pilot tested in a small sample (N = 5) of older cancer patients to judge phrasing and comprehension in older adults, but the complete SAKK C-SGA has not been assessed for ease of administration in daily oncological settings. To address this gap the aim of the current study was to evaluate feasibility and practical use of the SAKK C-SGA in Swiss clinical practices caring for older cancer patients.

**Table 1:** Content and operationalization of the SAKK cancer-specific geriatric assessment (C-SGA)

**Assessment domain**	**Assessment tool**	**Number of questions**	**How administered**	**Estimated time required (min)**	**Range: cut-off**
***Individual domains***					
Comorbidity	Age-adjusted Charlson Comorbidity Index (CCI)[27], [28]	18	Medical Record Abstraction (MRA)	5-10	0-43: ≥4
Function	Vulnerable Elders Survey (VES-13) [29]	12	Self-report or Interviewer Administered	<5	0-10: ≥3
Psychosocial	Geriatric Depression Scale 5-item short form (GDS-5) [30]	5	Self-report or Interviewer	<5	0-5: ≥2
	Modified MOS- Social Support Survey (mMOS-SS) [33]	8	Administered	<5	0-8: ≤2.5
Nutrition	Mini Nutritional Assessment (MNA) [31]	3	Interviewer administered and MRA	<4	0-14: ≤11
Cognition	Mini-Cog [32]	3	Interviewer administered	5	1-3 w/clock draw: 0 or 1–2 w/abnormal clock
***SAKK C-SGA***					
5 Domains	Six Measures: CCI, VES-13, GDS-5, mMOS-SS, MNA, Mini-Cog	22– Patient 20– MRA	Self-report, Interviewer Administered, MRA	<30	0-5: ≥3

## Methods

The protocol for this study was reviewed and approved by the Kantonsspital Graubünden (KSGR) Institutional Review Board (IRB) Ethics Committee and the Kantonsspital St. Gallen (KSSG) IRB Ethics Committee. The study was conducted in compliance with all federal regulations governing the protection and privacy of human subjects, the Helsinki Declaration, and with the informed consent of the participants.

### Study population

We conducted a cross sectional study of older cancer patients (total study population, N = 51) cared for at two oncology departments in canton hospitals in Switzerland: KSGR (N = 25) and KSSG (N = 26). The study population included a consecutive case series of older adults (**≥**65 years old) with physician permission to participate, a pathologically confirmed cancer diagnosis (newly diagnosed or relapsed), presenting for initiation of a new chemotherapy treatment (first-line or subsequent treatment) between July 2009 and March 2011.

### Data collection

Data for this study was collected using three instruments, the SAKK C-SGA plus physician and patient evaluation forms. The SAKK C-SGA (English, German, French, Italian) and scoring instructions (English only) are available by author request.

#### SAKK C-SGA

The SAKK C-SGA was administered in two parts by trained study personnel (hereafter referred to as physician) before the start of chemotherapy treatment. The interviewer-administered C-SGA patient questionnaire contained 22-questions with a variety of responses (e.g. rating scales, yes/no, word recall, clock drawing and spaces for recording the results of the MRA see Additional file 1). The C-SGA MRA included 20-questions for extracting health-related information (height, weight, comorbidities) from patient medical records.

#### Patient and physician evaluation forms

The patient evaluation form was filled-out by patients immediately after completion of the SAKK C-SGA patient interview. It had a total of 11-questions divided into two parts: (1) five patient information questions (gender, marital status, education, nationality, mother tongue); and (2) six questions relating to the patient questionnaire (estimated/acceptable time to complete, ease of administration, patient reaction, difficult/unanswered questions).

Personnel administering the SAKK C-SGA completed the physician evaluation immediately after each patient assessment. The physician questionnaire included 18-questions divided into four sections: (1) six patient information questions (age, general health, type cancer, treatment approach, life expectancy, performance status); (2) six questions relating to the C-SGA patient questionnaire (estimated/acceptable time to complete, ease of administration, patient reaction, difficult/unanswered questions); (3) four questions relating to MRA (estimated time to complete, ease of conducting, difficulty of obtaining information, missing information), and (4) two questions relating to training (years of experience, type of training).

### Analytic variables

#### Socio-demographic characteristics

We categorized information on age (65–69,70-79, 80+ years); gender (male, female); education (compulsory-up to 10th grade, secondary-high school, teacher training or vocational diploma; tertiary-undergraduate, graduate, post-graduate degree); marital status (single, married, widowed, divorced); and nationality (Swiss, other).

#### Health-related characteristics

Physician-rated general health was assessed by a single question with five answer likert scale ranging from “excellent” to “poor”. We also classified information on type of cancer (bladder, breast, colon, leukemia, lung, lymphoma, pancreatic, prostate, uterine, other); type of treatment (curative, palliative), physician judgement of life expectancy (<1 year, 1-2 years, 3 + years); and WHO performance score (0–4) with higher scores indicating worse health [35].

#### SAKK C-SGA

Table 1 shows content and operationalization of the SAKK C-SGA and individual domain assesment tools. SAKK C-SGA scoring was calculated as five individual domain scores (function, psychosocial, nutrition, cognition, comorbidity based on the published scoring rules for individual measures) and one overall C-SGA score. All scores were based on whole number ranges. The five individual domain scores were dichotomized as deficit or not based on literature-based pre-determined cut-off scores for each measure (see Table 1). Individual domains with more than one measure (e.g. psychosocial) were considered to be a deficit if at least one measure crossed the cut-off. The overall C-SGA score was calculated by summing the number of individual domain deficits (range 0–5) and dichotomizing deficits as ≤2 (fit for standard treatment) *vs.* ≥3 (unfit for standard treatment) [11,14].

#### SAKK C-SGA evaluation

The SAKK C-SGA was assessed by patient and physician estimated time to complete (mean time, acceptable yes/no), ease of completing (easy/fairly easy, just right, hard/very hard), patient reaction (interested, indifferent, rejecting), and difficult or unanswered questions (yes/no).

### Analytic methods

We examined the descriptive characteristics in the total population and compared the distributions between study sites using Student’s t-test for continuous variables and chi-square or Fisher’s exact tests for categorical variables testing for statistical significance in their differences. Similar methods were applied to results from the patient and physician evaluations. Associations between C-SGA scores and health-related characteristics were assessed using Spearman correlations. All analyses were performed using SAS (V9.3 SAS Institute, Cary, NC) and all p values were from two-sided tests.

## Results

### Population characteristics

Table 2 shows the characteristics of the individual study site populations as well as total study population. Most of the study population was Swiss (88%) and over half were aged 70–79 years, male with excellent/good physician-rated general health. Lung cancer (20%) and lymphoma (25%) were the most common types of cancers being treated in the total study population.

**Table 2:** Characteristics of the individual site and total study population

	**KSGR**	**KSSG**	**Total population**	
	***N = 25***	***N = 26***	***N = 51***	
**Characteristic**	**n (%)**			P value*
***Socio-demographic***				
Study site				
KSGR	25 (100)	–	25 (49)	
KSSG	–	26 (100)	26 (51)	
Age				
65–69 years	6 (24)	7 (27)	13 (25)	*0.57*
70–79 years	15 (60)	12 (46)	27 (53)	
80+ years	4 (16)	7 (27)	11 (22)	
Gender				
Male	17 (68)	11 (42)	28 (55)	*0.07*
Female	8 (32)	15 (58)	23 (45)	
Education				
Compulsory	7 (28)	5 (21)	12 (24)	*0.60*
Secondary	17 (68)	16 (67)	33 (67)	
Tertiary	1 (4)	3 (12)	4 (8.2)	
Marital status				
Single	0 (0)	0 (0)	0 (0)	*0.92*^*#*^
Married	18 (72)	17 (65)	35 (69)	
Widowed	7 (28)	7 (27)	14 (27)	
Divorced	0 (0)	2 (7.7)	2 (3.9)	
Nationality				
Swiss	22 (88)	23 (88)	45 (88)	*1.00*
Other	3 (12)	3 (12)	6 (12)	
***Health-related***				
Physician-rated general health				
Excellent/good	17 (71)	8 (32)	25 (51)	*0.007*
Fair	6 (25)	9 (36)	15 (31)	
Poor/very Poor	1 (4.1)	8 (32)	9 (18)	
Type of cancer				
Bladder	1 (4.0)	0 (0)	1 (1.9)	*0.40*^*#*^
Breast	0 (0)	1 (3.8)	1 (1.9)	
Colon	6 (24)	0 (0)	6 (12)	
Leukemia	0 (0)	7 (27)	7 (14)	
Lung	5 (20)	5 (19)	10 (20)	
Lymphoma	5 (20)	8 (31)	13 (25)	
Pancreatic	0 (0)	1 (3.8)	1 (1.9)	
Prostate	3 (12)	1 (3.8)	4 (7.8)	
Uterine	0(0)	1 (3.8)	1 (1.9)	
Other	5 (20)	2 (7.7)	7 (14)	
Type of treatment				
Curative	12 (48)	3 (12)	15 (29)	*0.006*
Palliative	13 (52)	23 (88)	36 (71)	
Life expectancy				
<1 year	4 (19)	16 (61)	20 (42)	*0.01*
1-2 years	8 (38)	6 (23)	14 (30)	
3+ years	9 (43)	4 (15)	13 (28)	
WHO performance score				
0	15 (60)	4 (15)	19 (37)	*<0.001*
1	9 (36)	9 (35)	18 (35)	
2	1 (4.0)	13 (50)	14 (27)	

KSGR Kantonsspital Graubünden, KSSG Kantonsspital St. Gallen, WHO World Health Organization.

* Test of difference between study sites.

^#^ Significance test excluding marital status single and cancers with zero cells.

The individual study site populations were evenly distributed (KSGR 49%, KSSG 51%) but differed on health-related characteristics. KSGR had more patients with excellent/good (71% *vs.* 32%, p = 0.007) physician-rated general health. The majority of KSSG patients were receiving palliative treatment (88% *vs.* 52%, p = 0.006); had a life expectancy of <1 year (61% *vs.* 19%, p = 0.01); and a WHO performance score of 2 (50% *vs.* 4%, p = <0.001).

### Patient and physician evaluation of the SAKK C-SGA

Table 3 displays the results of patient and physician evaluation of the SAKK C-SGA. The time to complete the patient questionnaire was considered acceptable by almost all patients and physicians. Patients reported slightly shorter times to complete the questionnaire than physicians (17.33 ± 7.34 *vs.* 20.59 ± 6.53 minutes, p = 0.02). Both groups rated the SAKK C-SGA patient questionnaire as easy/fairly easy to complete (91% *vs.* 84% respectively, p = 0.14) with few difficult or unanswered questions. Physicians reported the MRA took on average 8.32 ± 4.72 minutes, 100% considered the time to complete acceptable with 96% rating it as easy/fairly easy to complete. The majority of personnel administering the SAKK C-SGA were nursing staff with over 11 years of clinical experience (data not shown).

**Table 3:** Patient and physician evaluation of SAKK cancer-specific geriatric assessment (C-SGA)

	**Patient *N = 51***	**Physician *N = 9***	
	**n (%) or mean ± SD**		P value*
***Patient questionnaire***			
Estimated time to complete			
Average time in minutes	17.33 ±7.34	20.59 ±6.53	*0.02*
Rated time as acceptable	44 (96)	51 (100)	*0.22*
Ease of completing			
Easy/fairly easy	42 (91)	43 (84)	*0.14*^*#*^
Just right	4 (8.7)	3 (5.9)	
Hard/very hard	0 (0)	5 (9.8)	
Patient reaction			^*§*^
Interested	37 (73)	44 (86)	
Indifferent	7 (14)	5 (9.8)	
Rejecting	0 (0)	2 (3.9)	
Difficult to answer questions			
Yes	5 (11)	8 (16)	*0.91*
No	39 (89)	43 (84)	
Unanswered questions			
Yes	6 (14)	11 (22)	*0.03*
No	38 (86)	40 (78)	
***Medical record abstraction***			
Estimated time to complete			
Average time in minutes	–	8.32 ±4.72	
Rated time as acceptable	–	50(100)	
Ease of completing			
Easy/fairly easy	–	47 (96)	
Just right	–	2 (4.1)	
Hard/very hard	–	0 (0)	

*C-SGA* Cancer-Specific Geriatric Assessment; *SD* standard deviation.

* Test of difference between patients and physicians.

^#^ Significance test based on two categories created by collapsing the bottom two categories with the smallest cell counts into one (e.g. Easy/Fairly easy vs. Just right/Hard/Very hard).

^§^ Significance test not possible due to zero cells.

### C-SGA characteristics of the study population

Table 4 describes the C-SGA characteristics of the study population. The overall mean C-SGA score was 2.4 ± 1.12. Patients at KSGR had lower C-SGA scores (2.00 ± 1.19 *vs.* 2.81 ± 0.90, p = 0.009) and a smaller proportion (28% *vs.*65%, p = 0.008) was above the C-SGA cut-off score compared to KSSG. In the total population the most common domain deficits were function, nutrition and comorbidity. By site population KSGR had statistically significantly fewer patients with a nutrition domain deficit than KSSG. C-SGA score was correlated with all health-related characteristics but not with age. Health-related characteristics differed by C-SGA cut-off scores. More patients with C-SGA score below the cut-off had excellent/good physician-rated general health (67% *vs.* 32%, p = 0.007); were receiving curative treatment (44% *vs.* 13%, p = 0.02); and had a WHO performance score of 0 or 1 (85% *vs.* 58%, p = 0.002).

**Table 4:** Cancer-specific geriatric assessment (C-SGA) characteristics in the individual site and total study populations

***C-SGA characteristics by study site and total population***				
	**KSGR**	**KSSG**	**Total population**	
	***N = 25***	***N = 26***	***N = 51***	
	**n (%)**			P value*
Mean C-SGA score	2.00 ±1.19	2.81 ±0.90	2.4 ±1.12	*0.009*
C-SGA over cut-off score				
Yes	7 (28)	17 (65)	24 (47)	*0.008*
No	18 (72)	9 (35)	27 (53)	
C-SGA by domain deficits				
Function				
Yes	14 (56)	18 (69)	32 (63)	*0.33*
No	11 (44)	8 (31)	19 (37)	
Psychosocial				
Yes	1 (4.0)	7 (27)	8 (16)	*0.05*
No	24 (96)	19 (73)	43 (16)	
Nutrition				
Yes	13 (52)	22 (85)	35 (69)	*0.02*
No	12 (48)	4 (15)	16 (31)	
Cognition				
Yes	3 (12)	1 (3.9)	4 (7.8)	*0.35*
No	22 (88)	25 (96)	47 (92)	
Comorbidity				
Yes	19 (76)	25 (96)	44 (86)	*0.05*
No	6 (24)	1 (3.9)	7 (14)	
***C-SGA association with health-related characteristics***				
	**Correlation coefficient**		***P value***	
Age	0.12		*0.43*	
Physician-rated general health	0.39		*0.008*	
Type of treatment	−0.38		*0.01*	
Life expectancy	−0.32		*0.03*	
WHO performance score	0.63		*<0.0001*	
***Health-related characteristics by C-SGA cut-off score***				
	**Below C-SGA cut-off score**		**Above C-SGA cut-off score**	
	***N = 27***		***N = 24***	
	**n (%)**			***P value***^***#***^
Age				
65–69 years	8 (30)		5 (21)	*0.72*
70–79 years	14 (52)		13 (54)	
80+ years	5 (18)		6 (25)	
Physician-rated general health				
Excellent/good	18 (67)		7 (32)	*0.007*
Fair	8 (30)		7 (32)	
Poor/very poor	1 (3.7)		8 (36)	
Type of treatment				
Curative	12 (44)		3 (13)	*0.02*
Palliative	15 (56)		21 (87)	
Life expectancy				
<1 year	7 (30)		13 (54)	*0.16*
1–2 years	7 (30)		7 (29)	
3+ years	9 (39)		4 (17)	
WHO performance score				
0	16 (59)		3 (12)	*0.002*
1	7 (26)		11 (46)	
2	4 (15)		10 (42)	

*C-SGA* Cancer-Specific Geriatric Assessment, *KSGR* Kantonsspital Graubünden *KSSG* Kantonsspital St. Gallen, *SD* Standard Deviation, *WHO* World Health Organization.

* Test of difference between study sites.

^#^ Test of difference between groups by C-SGA cut-off.

## Discussion

This study demonstrated that the SAKK C-SGA is feasible and easy to implement in daily clinical practice. The overall time to complete was less than 30 minutes and considered acceptable by patient and physician alike. Importantly, most participants rated the SAKK C-SGA (patient questionnaire and MRA) as easy or fairly easy to complete. Only a small number of patients or physicians reported questions that were either difficult or unanswered. This likely reflects real-world patient-specific difficulties encountered in daily oncological practice as opposed to problems with the questions themselves. This is supported by the fact that all measures included in the SAKK C-SGA are widely used and previously validated. Plus there was no pattern to the individual questions that were reported as difficult or unanswered (e.g. only one question was mentioned twice, questions identified were not domain-specific).

These findings also suggest that the SAKK C-SGA was able to objectively discriminate older patients’ health. The difference in SAKK C-SGA scores between study sites mirrored differences in the site-specific patient characteristics. KSGR had an overall healthier population (as assessed by other health-related measures) with a higher proportion of patients being treated for curative intent than KSSG. Correspondingly, KSGR SAKK C-SGA scores were lower and a higher proportion was below the cut-off score (i.e. fit for standard treatment). In both sites more patients below the cut-off score had better physician-rated general health, a longer life expectancy as well as better WHO performance scores. Interestingly, in this population C-SGA scores were related to other health measures but not age, underscoring the potential advantage of C-SGA versus age-based decision-making. As expected (i.e. health-related measures were designed to assess unique health states) there was not complete overlap in how individual health-related measures and the SAKK C-SGA identified the patient population. This is similar to findings by other researchers who found physicians and C-SGA do not identify the same patient populations as fit/unfit for treatment and that C-SGA compares with but is not identical to assessments based other health-measures [36-40].

Although shorter screening tools exist and the SAKK C-SGA had high correlation with other simpler health-related measures (e.g. WHO performance score) it affords additional clinical benefit to patient and provider [4,25,40-44]. The time to complete though longer than a brief screen is suitable for pre-treatment or pre-trial oncological work-ups. The time to complete the SAKK C-SGA in daily oncological practice was similar to that of another C-SGA tool pilot tested in clinical trial settings [25]. It also requires much less time and no referral for a full comprehensive geriatric assessment. This is particularly important since not all healthcare systems offer specialized geriatric care. Second, the SAKK C-SGA identifies individual domain deficits for intervention acknowledged within the C-SGA literature as having specific benefits in the care of older cancer patients [2,16,19,45]. The problems identified can be addressed either within oncological practices and/or by referral depending on available resources and expertise. For example, engaging social workers, arranging transportation, or providing nutritional counseling before start of treatment could be arranged by staff handling cancer treatment, general practitioners, or referral to a geriatrician depending on individual patient needs and care situations.

The key advantage is that such interventions, regardless of where they are initiated, may mitigate an older patient’s risk for poor cancer treatment outcomes and increase their quality of life. A recent study in Spain showed that C-SGA detects more information than oncological evaluation alone [46]. Another in Canada found that in 70% of their study patients C-SGA identified previously unidentified medical problems [47]. In this Swiss population, over half of our patients were identified by the SAKK C-SGA as not a risk for poor outcomes (i.e. below cut-off/fit for standard treatment). Nevertheless, all but one of these patients had a deficit in at least one domain that otherwise may not have been identified by oncological evaluation alone or even a briefer C-SGA screen. Thus use of the SAKK C-SGA provides readily usable information that can improve outcomes for patients above or below the cut-off without delay (i.e. no additional assessment necessarily required). However, we did find that when dementia was present the SAKK C-SGA (like any geriatric assessment) was challenging to administer. In patients with dementia (especially advanced cases) decision-making regarding treating cancer is likely not be aided by objective C-SGA measurement. But instead will require a more complex individualized process between patient-physician-family/caregiver.

Other researchers in the field and SIOG have identified the need for shorter C-SGA tools applicable for busy clinical oncology settings [48]. The SAKK C-SGA has several benefits directly addressing this need. First, assessment using the SAKK C-SGA requires much less time than a full geriatric assessment and does not require referral to a specialist or geriatrics training to administer. Second, using standard geriatric assessment tools in key domains the SAKK C-SGA maximizes information gathering and minimizes patient/physician burden. In fact, since the tool can be administered by any combination of patient (all but Mini-Cog), trained staff, or physician it is easily customized to the individual patient and clinical setting. Lastly, our findings suggest that use of the SAKK C-SGA is feasible in clinical practice and may be well suited to determine eligibility for clinical trials based on patient health instead of chronological age.

A major challenge of C-SGA is to find a balance between time to conduct and producing clinically useful information (i.e. identifying targets for intervention). The SAKK C-SGA is a step forward in this balancing act but can be further improved. This study used an electronic excel-based CCI calculator that made collecting comorbidity data and calculating CCI information easier, more accurate, and immediately available [28]. Based on our positive experience with the excel-based CCI we decided that an electronic version of the SAKK C-SGA could offer similar advantages in clinical practice and clinical trials. An electronic SAKK C-SGA would make gathering data more efficient, produce real time results that can be immediately incorporated into treatment planning, and increase the likelihood of more widespread and uniform use. Development is underway and validation and feasibility studies are planned.

Several strengths and weaknesses of this study should be considered. The SAKK C-SGA was developed with input from geriatricians and oncologists specifically for use in busy oncological and clinical trial settings. The tool includes only standard psychometrically evaluated geriatric assessment measures covering previously identified key domains. The SAKK C-SGA is easy to score and available in multiple languages. However, the results of this study are based on a small number of patients in Switzerland. Thus generalizability of these findings to other clinical settings and other healthcare systems are limited. We did not require exact measurement of time to complete and assume that most patients/physicians estimated times. However, both the patient and physician estimates had similar standard deviations and the perception of time to complete (i.e. estimated time was acceptable to nearly all) is an important factor in willingness to adopt the tool. The SAKK C-SGA should be further tested in larger patient samples, a variety of settings, and over longer periods of time to include outcome data.

## Conclusions

In conclusion, these results suggest that the SAKK C-SGA is a promising tool for use in clinical practice. It demonstrated discriminative ability based on objective geriatric assessment measures. It also provides important clinical information that could be used for interventions aimed at optimizing outcomes in older cancer patients. Future studies of SAKK C-SGA reliability, discriminative ability for clinical decision-making based on treatment outcomes, accuracy predicting cancer-related outcomes, ability to improve outcomes, and generalizability are warranted.

## Abbreviations

CCI: Charlson comorbidy index; C-SGA: Cancer-specific geriatric assessment; GDS: Geriatric depression scale; IRB: Institutional review board; KSGR: Kantonspital graubünden; KSSG: Kantonspital St. Gallen; mMOS-SS: Modified medical outcomes study -social support survey; MNA: Mini nutritional assessment; MRA: Medical record abstraction; SAKK: Schweizerische arbeitsgemeinschaft für klinische krebsforschung; SIOG: International society of geriatric oncology; VES: Vulnerable elders survey; WHO: World Health Organization.

## Competing interests

None of the authors have a conflict of interest. The sponsors had no role in the design, methods, subject recruitment, data collection, analysis, or paper preparation.

## Authors’ contributions

All authors made substantial contributions to this work as follows: study design- KC, PB, FH, UM. Data collection- RC, MF, GR, FH, UM. Data analysis- LN, AS, KC. Interpretation of data- all; manuscript preparation and approval of drafts and final submitted version- all.

## Pre-publication history

The pre-publication history for this paper can be accessed here:

http://www.biomedcentral.com/1472-6947/13/93/prepub

## Supplementary Material

Additional file 1 Detailed contents of the SAKK Cancer-Specific Geriatric Assessment (C-SGA). Click here for file
