# Supplementary material for: The SAKK cancer-specific geriatric assessment (C-SGA): a pilot study of a brief tool for clinical decision-making in older cancer patients
Source: BMC Med Inform Decis Mak. 2013 Aug 23;13:93. doi: 10.1186/1472-6947-13-93 (PMC3765229; doi:10.1186/1472-6947-13-93)
Supplement: Additional file 1 — Detailed contents of the SAKK Cancer-Specific Geriatric Assessment (C-SGA). [file 1472-6947-13-93-S1.docx]

| Table 2: Detailed contents of the SAKK Cancer-Specific Geriatric Assessment (C-SGA) | | | | | | | | | | | | | | | | | | | | | |
| --- | --- | --- | --- | --- | --- | --- | --- | --- | --- | --- | --- | --- | --- | --- | --- | --- | --- | --- | --- | --- | --- |
| **Medical Record Abstract (MRA)**  **Charlson Comorbidty Index (CCI)**  __________ un-age-adjusted ___________ age-adjusted  Note: Calculate using reference 28  **Body Mass Index (BMI)** __________ = weight(kg) / height²(m²)  __________ weight in kg __________ height in cm | | | | | | | | | | | | | | | | | | | | | |
| **Patient Interview**  **Vulnerable Elders Survey (VES-13)** | | | | | | | | | | | | | | | | | | | | | |
|  | | | | | Poor | | | Fair | | | | Good | | Very Good | | | | | Excellent | | |
| 1. In general, compared to other people your age, would you say that your health is: | | | | | 1 | | | 1 | | | | 0 | | 0 | | | | | 0 | | |
| 1. How much difficulty, on average, do you have with the following physical activities: | | | | | | | | | | | | | | | | | | | | | |
|  | | No difficulty | | | | A little difficulty | | | | Some difficulty* | | | A lot of difficulty* | | | | | Unable to do* | | | |
| 1. Stooping, crouching or kneeling? | | 0 | | | | 0 | | | | 1 | | | 1 | | | | | 1 | | | |
| 1. Lifting, or carrying objects as heavy as 10 pounds? | | 0 | | | | 0 | | | | 1 | | | 1 | | | | | 1 | | | |
| 1. Reaching or extending arms above shoulder level? | | 0 | | | | 0 | | | | 1 | | | 1 | | | | | 1 | | | |
| 1. Writing, or handling and grasping small objects? | | 0 | | | | 0 | | | | 1 | | | 1 | | | | | 1 | | | |
| 1. Walking a quarter of a mile? | | 0 | | | | 0 | | | | 1 | | | 1 | | | | | 1 | | | |
| 1. Heavy housework such as scrubbing floors or washing windows? | | 0 | | | | 0 | | | | 1 | | | 1 | | | | | 1 | | | |
| 1. Because of your health or a physical condition, do you have any difficulty: | | | | | | | | | | | | | | | | | | | | | |
| 1. Shopping for personal items (like toilet items or medicine)? | Yes | | | → | | Do you get help with shopping? | | | | | | | | | | Yes | | | | | No |
|  | No | | |  | |  | | | | | | | | | |  | | | | |  |
|  | Don’t do | | | → | | Is that because of your health? | | | | | | | | | | Yes | | | | | No |
| 1. Managing money (like keeping track of expenses or paying bills)? | Yes | | | → | | Do you get help with managing money? | | | | | | | | | | Yes | | | | | No |
|  | No | | |  | |  | | | | | | | | | |  | | | | |  |
|  | Don’t do | | | → | | Is that because of your health? | | | | | | | | | | Yes | | | | | No |
| 1. Walking across the room? USE OF CANE OR WALKER IS OKAY | Yes | | | → | | Do you get help with walking? | | | | | | | | | | Yes | | | | | No |
|  | No | | |  | |  | | | | | | | | | |  | | | | |  |
|  | Don’t do | | | → | | Is that because of your health? | | | | | | | | | | Yes | | | | | No |
| 1. Doing light housework (like washing dishes, straightening up, or light cleaning? | Yes | | | → | | Do you get help with light housework? | | | | | | | | | | Yes | | | | | No |
|  | No | | |  | |  | | | | | | | | | |  | | | | |  |
|  | Don’t do | | | → | | Is that because of your health? | | | | | | | | | | Yes | | | | | No |
| 1. Bathing or showering? | Yes | | | → | | Do you get help with bathing or showering? | | | | | | | | | | Yes | | | | | No |
|  | No | | |  | |  | | | | | | | | | |  | | | | |  |
|  | Don’t do | | | → | | Is that because of your health? | | | | | | | | | | Yes | | | | | No |
|  |  | | |  | |  | | | | | | | | | |  | | | | |  |
|  |  | | |  | |  | | | | | | | | | |  | | | | |  |
| **Modified Medical Outcomes Study Social Support Survey (mMOS-SS)** | | | | | | | | | | | | | | | | | | | | | |
| *People sometimes look to others for companionship, assistance or other types of support. I’d like you to tell me how often each of the following kinds of support are available to you if you need it?*  If you needed it, how often is someone available… | | | | | | | | | | | | | | | | | | | | | |
|  | | | None of the time | | | | A little of the time | | | | Some of the time | | | | Most of the time | | | | | All of the time | |
| 1. …to help you if you were confined to bed | | | 1 | | | | 2 | | | | 3 | | | | 4 | | | | | 5 | |
| 1. …to take you to the doctor if you need it | | | 1 | | | | 2 | | | | 3 | | | | 4 | | | | | 5 | |
| 1. …to have a good time with | | | 1 | | | | 2 | | | | 3 | | | | 4 | | | | | 5 | |
| 1. …to prepare your meals if you are unable to do it yourself | | | 1 | | | | 2 | | | | 3 | | | | 4 | | | | | 5 | |
| 1. …to help with daily chores if you were s | | | 1 | | | | 2 | | | | 3 | | | | 4 | | | | | 5 | |
| 1. …to turn to for suggestions about how to deal with a personal problem | | | 1 | | | | 2 | | | | 3 | | | | 4 | | | | | 5 | |
| 1. …who understands your problems | | | 1 | | | | 2 | | | | 3 | | | | 4 | | | | | 5 | |
| 1. …to love and make you feel wanted | | | 1 | | | | 2 | | | | 3 | | | | 4 | | | | | 5 | |
|  |  | | |  | |  | | | | | | | | | |  | | | | |  |
| **Geriatric Depression Scale (GDS-5)** | | | | | | | | | | | | | | | | | | | | | |
| 1. Are you basically satisfied with your life? | | | | | | | | | | 0 = Yes | | | | | | | 1= No | | | | |
| 1. Do you often get bored? | | | | | | | | | | 0 = Yes | | | | | | | 1= No | | | | |
| 1. Do you often feel helpless? | | | | | | | | | | 0 = Yes | | | | | | | 1= No | | | | |
| 1. Do you prefer to stay at home, rather than going out and doing new things? | | | | | | | | | | 0 = Yes | | | | | | | 1= No | | | | |
| 1. Do you feel pretty worthless the way you are now? | | | | | | | | | | 0 = Yes | | | | | | | 1= No | | | | |
|  |  | | |  | |  | | | | | | | | | |  | | | | |  |
| **Mini Nutritional Assessment (MNA)** | | | | | | | | | | | | | | | | | | | | | |
| 1. Has food intake declined over the past 3 months due to loss of appetite, digestive problems, chewing or swallowing difficulties? | | | | | | | | | 0 = severe loss of appetite  1 = moderate loss of appetite  2 = no loss of appetite | | | | | | | | | | | | |
| 1. Weight loss during the last 3 months | | | | | | | | | 0 = weight loss greater than 3kg (6.6lbs)  1 = does not know  2 = weight loss between 1 & 3kg (2.2lbs & 6.6lbs)  3 = no weight loss | | | | | | | | | | | | |
| 1. Has suffered psychological stress or acute disease in the past 3 months | | | | | | | | | 1= Yes  0 = No | | | | | | | | | | | | |
| ***Optional Questions:***  Mobility (can use VES-13 instead) | | | | | | | | | 0 = bed or chair bound  1 = able to get out of bed/chair but does not go out  2 = goes out | | | | | | | | | | | | |
| Neuropsychological problems (can use GDS-5 and MiniCog instead) | | | | | | | | | 0 = severe dementia or depression  1 = mild dementia  2 = no psychological problems | | | | | | | | | | | | |
|  |  | | |  | |  | | | | | | | | | |  | | | | |  |
| **Mini Cog** | | | | | | | | | | | | | | | | | | | | | |
| 1. I would like to test your memory. Can you repeat the words I said? | | | "ball” “car” “man" | | | | | | | | | | | | | | | | | | |
| 1. Inside the circle draw the hours of a clock as if a child would draw them. Place the hands of the clock to represent the time “forty five minutes past ten o’clock” | | |  | | | | | | | | | | | | | | | | | | |
| 1. Recall: What were the three words I asked you to say earlier? | | | ____________ _____________ _____________ | | | | | | | | | | | | | | | | | | |
|  |  | | |  | |  | | | | | | | | | |  | | | | |  |
